# NQO1 protein expression predicts poor prognosis of non-small cell lung cancers

**DOI:** 10.1186/s12885-015-1227-8

**Published:** 2015-03-31

**Authors:** Zhenling Li, Yue Zhang, Tiefeng Jin, Jiguang Men, Zhenhua Lin, Peng Qi, Yingshi Piao, Guanghai Yan

**Affiliations:** 1 Department of Pathology & Cancer Research Center, Yanbian University Medical College, Yanji, 133002 China; 2 Department of TCM, Jilin Cancer Hospital, Changchun, 130012 China; 3 Department of Anatomy and Histology and Embryology, Yanbian University Medical College, Yanji, 133002 China; 4 Department of Pathophysiology, Yanbian University Medical College, Yanji, 133002 China

**Keywords:** Non-small cell lung cancer, NQO1, Immunohistochemistry, Prognosis, Survival analysis

## Abstract

**Background:**

High-level expression of NAD(P)H: quinoneoxidoreductase 1 (NQO1) has been correlated with many types of human cancers, suggesting that NQO1 plays important roles in tumor occurrence and progression. This study attempted to explore the role of NQO1 in tumor progression and prognostic evaluation of non-small cell lung cancer (NSCLC).

**Methods:**

Total 164 tissue samples, including 150 NSCLC paired with the adjacent non-tumor tissues and 14 normal lung tissues, were picked-up for immunohistochemical (IHC) staining of the NQO1 protein, and immunofluorescence (IF) staining was also performed to detect the subcellular localization of the NQO1 protein in A549 human lung cancer cells. The correlation between NQO1 expression and clinicopathological characteristics were evaluated by Chi-square test and Fisher’s exact tests. The disease-free survival (DFS) and overall survival (OS) rates of NSCLC patients were calculated by the Kaplan-Meier method, and univariate and multivariate analyses were performed using the Cox proportional hazards regression model.

**Results:**

The NQO1 protein showed a mainly cytoplasmic staining pattern in lung cancer cells, including adenocarcinoma and squamous cell carcinoma (SCC). Both positive rate and strongly positive rate of NQO1 protein expression were significantly higher in NSCLC (59.3% and 28.0%) than that in adjacent non tumor (8.0% and 1.3%) and normal lung tissues (0%). The positive rate of NQO1 was related with clinical stage and lymph node metastasis, and the strongly positive rate of NQO1 protein was significantly correlated with tumor size, poor differentiation, advanced clinical stage and lymph node metastasis in NSCLC. Additionally, survival analyses showed that the patients with NQO1 positive expression had lower OS rates compared with those with NQO1 negative expression in the groups of T1-2, T3-4, without LN metastasis and stage I-II of NSCLC, respectively; however, in the groups of patients with LN metastasis or III-IV stages, OS rate was not correlated with NQO1 expression status. Moreover, multivariate analysis suggested that NQO1 emerged as a significant independent prognostic factor along with tumor size, differentiation, lymph node metastasis and clinical stage in patients with NSCLC.

**Conclusions:**

NQO1 is upregulated in NSCLC, and it may be a useful poor prognostic biomarker and a potential therapeutic target for patients with NSCLC.

## Background

Non-small cell lung cancer (NSCLC) accounted for approximately 85% of all lung cancers, and it is the most common cause of death in both men and women [1]. Currently, molecular target therapy is one of the promising field of NSCLC treatment, and its target includes epidermal growth factor receptor (EGFR) and echinoderm microtubule associated protein like4-anaplastic lymphoma kinase (EML4-ALK). EGFR tyrosine kinase inhibitor (EGFR TKI, such as gefitinib and erlotinib) and EML4/ALK inhibitor (Crizotinib) have achieved better results in the clinical therapy of advanced NSCLC [2,3]. Despite progress in the multimodality treatment of lung cancer, prognosis is still poor, with 10-15% 5-year survival rates. More than 90% of deaths from NSCLC are attributable to metastases [1,4].

NAD(P)H: quinone oxidoreductase 1 (NQO1, EC 1.6.99.2) is well known as DT-diaphorase, and it can protect cells against radiation and chemical-induced oxidative stress. NQO1 is a cytosolic flavoenzyme that catalyzes the obligatory two-electron reduction of a variety of quinone substrates by using NADH or NADPH as electron donors [5]. And several functions of NQO1 have been found, such as xenobiotic detoxification, superoxide scavenging, modulation of p53, maintenance of endogenous antioxidants, and proteasomal degradation [6]. Due to the ability of NQO1, it is imaginable that NQO1 may play an important role in protecting normal cells against oxidative damage and electrophilic attack [7,8]. Recent studies reported that NQO1 is mainly expressed in cytosol, and low expression levels have been found in the nucleus. Moreover, NQO1 was found to be expressed at high levels in many human cancers, including liver, colon, pancreas and cholangiocarcinoma [9-12]. Garate *et al*. [13] indicated that the expression of NQO1 protein significantly induced cell cycle progression and led to the proliferation of melanoma cells by the up-regulation of cyclin A2, B1 and D1. However, the role of NQO1 in progression of lungcancer cells remains unidentified, and the correlation between NQO1 expression and NSCLC has not been adequately elucidated yet.

To determine whether NQO1 is important in the tumorigenesis of NSCLC and investigate the prognostic value of NQO1 expression level, total 150 cases of NSCLC paired with the adjacent non-tumor tissues and 14 of normal lung tissues were selected for NQO1 IHC staining. Our data uncover that NQO1 is frequently upregulated in NSCLC compared with the normal counterpart, and suggest that NQO1 may be an independent biomarker for prognostic evaluation of patients with NSCLC.

## Methods

### Ethic statement

This research complied with the Helsinki Declaration and was approved by the Human Ethics Committee and the Research Ethics Committee of Yanbian University Medical College. Patients were informed that the resected specimens were stored by the hospital and potentially used for scientific research, and that their privacy would be maintained. Follow-up survival data were collected retrospectively through medical record analyses.

### Clinical samples

Total 164 tissue samples were used for this study, including 150NSCLC paired with the adjacent non-tumor tissues and 14 normal lung tissues (from autopsy cases). All of these tissues were collected from Shanghai Outdo Biotech Co. Ltd. (Outdo Biotech) and Tissue Bank of Yanbian University Medical College. All tissues were routinely fixed in 10% buffered formalin and embedded in paraffin blocks. The study protocol was approved by the institutional review board of Yanbian University Medical College. The pathological parameters, including gender, age, tumor size, clinical stage, differentiation, nodal metastasis and survival data, were carefully reviewed in all 150 NSCLC cases.

The patients with NSCLC including 112 males and 38 females, and ranging from 43 to 76 years with a mean age of 62 years. A total of 150 patients, 99 cases were 60 years old or over, and 51 cases were below 60 years old. All cases were confirmed with NSCLC by pathological examination. TNM staging was assessed according to the staging system established by the American Joint Committee on Cancer (AJCC). Of the 150 NSCLC, 98 cases were stages I-II while 52 cases were stages III-IV, and for the tumor sizes, 119 cases were defined as T1-T2 and 31 cases were T3-T4. In addition, 34 cases were defined as well differentiated, while 89 cases as moderately and 27 cases as poorly differentiated. Additionally, 96 cases have lymph node (LN) metastasis, and 54 cases have no LN metastasis. None of the patients received radio-chemotherapy before surgery. The 150 patients with NSCLC had been followed for eight years or until death. In this study, 150 cases of adjacent non-tumor lung tissues from the cancer resection margin and 14 cases of normal lung tissues were also included.

### Immunofluorescence (IF) staining for NQO1 protein in A549 lung cancer cells

Lung cancer cell line A549 was grown on coverslips to 70% confluence, then all cells were fixed with 4% paraformaldehyde for 10 minutes and permeabilized with 0.5% TritonX-100 for 10 minutes after 24 hours. Blocking was performed with 3% Albumin Bovine V (A8020, Solarbio, Beijing, China) for 1 hour at the room temperature (RT). After washing with PBS, cells were incubated with antibody against NQO1 (1:200, Cell Signaling Technology, Boston, USA) for 2 hours at 37°C, and followed the incubation by Alexa Fluor®488 goat anti-rabbit IgG (H + C) (A11008, Invitrogen, USA) respectively, for 1 hour at RT. After washing with PBS, cells were counterstained with 49-6-diamidino-2-phenylindole (DAPI) (C1006, Beyotime, Shanghai, China) and the coverslips were mounted with Antifade Mounting Medium (P0126, Beyotime, Shanghai, China). Finally, the immunofluorescence signals were visualized and recorded by Leica SP5II confocal microscope.

### Immunohistochemistry (IHC) for NQO1 protein in paraffin-embedded tissues

IHC analysis was performed using the DAKO LSAB kit (DAKO A/S, Glostrup, Denmark). Briefly, to eliminate endogenous peroxidase activity, 4 μm thick tissue sections were deparaffinized, rehydrated and incubated with 3% H_2_O_2_ in methanol for 15 min at RT. The antigen was retrieved at 95°C for 20 min by placing the slides in 0.01 M sodium citrate buffer (pH 6.0). The slides were then incubated with NQO1 antibody (1:600, BD Biosciences Pharmingen, CA, USA) at 4°C overnight. After incubation with biotinylated secondary antibody at RT for 30 min, the slides were incubated with streptavidin-peroxidase complex at RT for 30 min. IHC staining was developed by using 3,3′-diaminobenzidine, and Mayer’s hematoxylin was used for counterstaining. In addition, the positive tissue sections were processed with omitting of the primary antibody as negative controls.

### Evaluation of IHC staining

All specimens were examined by two investigators (Jin T & Lin Z) who did not possess knowledge of the clinical data. In case of discrepancies, a final score was established by reassessment on a double-headed microscope. Briefly, the IHC staining for NQO1 was semi-quantitatively scored as ‘-’ (negative, no or less than 5% positive cells), ‘+’ (5-50% positive cells), and ‘++’ (more than 50% positive cells, considered as strongly positive). Only the cytoplasmic expression pattern was considered as positive staining.

### Statistical analysis

Statistical analyses were performed using the SPSS software program for windows, version 17.0 (SPSS, Inc., Chicago, IL, USA). Correlation between NQO1 expression and clinicopathological characteristics were evaluated by Chi-square test and Fisher’s exact tests. The survival rates after tumor removal were calculated by the Kaplan-Meier method, and differences in survival curves were analyzed by the Log-rank tests. Multivariate survival analysis was performed on all the significant characteristics measured by univariate survival analysis through the Cox proportional hazard regression model. P-values less than 0.05 were considered statistically significant.

## Results

### High expression of NQO1 protein in NSCLC

**Figure 1:**
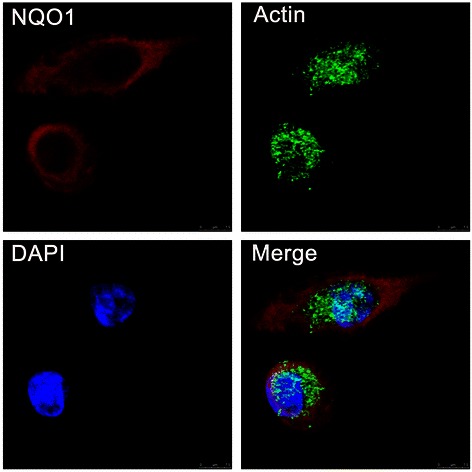
**IF staining for NQO1 protein in A549 human lung cancer cells.** NQO1 protein located in the cytoplasm of A549 cells (Red for NQO1, Green for Actin, and Blue for DAPI).

**Figure 2:**
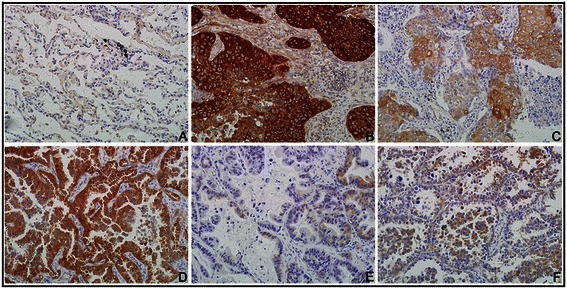
**IHC staining for NQO1 protein expression in lung tissues. (A)** NQO1 protein was negative in normal lung tissues. **(B)** NQO1 protein was showed diffuse and strong positive staining in cytopalsm of lung SCC cells with LN metastasis. **(C)** NQO1 was weakly positive in lung SCC without LN metastasis. **(D)** Diffuse and strong positive NQO1 protein signal in lung adenocarcinoma. **(E & F)** NQO1 protein staining is negative or weakly positive in lung adenocarcinoma. (Original magnification, 200× in **A-F**).

IF staining indicated that NQO1 protein was mainly located in the cytoplasm of A549 lung cancer cells (Figure 1). IHC staining consistently showed that the NQO1 protein was located in the cytoplasm of lung SCC and adenocarcinoma (Figure 2B & D). The positive rate of the NQO1 protein expression was 59.3% (89/150) in NSCLC tissues, which was significantly higher than that in adjacent non-tumor (8.0%, 12/150), and the expression were all negative in normal lung tissues (*P* < 0.01). Similarly, the strongly positive rate of NQO1 expression was 28.0% (42/150) in NSCLC, which was also significantly higher than that in adjacent non-tumor (1.3%, 2/150) (*P* < 0.01) (Table 1).

**Table 1:** **NQO1 protein expression in NSCLC**

Diagnosis	No. of cases	NQO1 protein expression			Positive rate(+ ~ ++)	Strongly positive rate(++)
		-	+	++		
NSCLC	150	61	47	42	59.3%**	28.0%**
Adjacent non tumor	150	138	10	2	8.0%	1.3%
Normal lung tissues	14	14	0	0	0	0

**P < 0.01compared with normal lung tissues and adjacent non tumor tissues.

### Clinicopathological significance of NQO1 expression in patients with NSCLC

The relationship between NQO1 protein and the clinicopathological parameter of NSCLC was analyzed. The positive rate of NQO1 protein was related with clinical stage and lymph node metastasis. Moreover, the strongly positiverate of NQO1 protein was significantly higher in NSCLC with T3-4 (>5 cm) tumor size than in cases with T1-2 (≤5 cm) tumor size (*P* = 0.005). Similarly, we found that the strongly positive rate of NQO1 protein was significantly higher in stages III-IV (36.54%, 19/52) than those in stages I-II (23.47%, 23/98) (*P* = 0.003). Also, it was higher in poorly differentiated NSCLC (55.56%, 15/27) than in moderately (31.46%, 28/89) and well differentiated NSCLC (26.47%, 9/34) (*P* = 0.012). Additionally, it was also higher in NSCLC patients with lymph node metastasis (50.00%, 27/54) than in cases without metastasis (15.63%, 15/96) (*P* = 0.000). However, there was no significant correlations between high-level NQO1 expression and gender, and age of patients with NSCLC (*P* > 0.05, respectively) (Table 2).

**Table 2:** **Correlation between NQO1 expression and clinicopathological features of NSCLC**

Variables	Case no.	NQO1 positive(+ ~ ++)		NQO1 strongly positive(++)	
		n (%)	*P*value	n (%)	*P*value
**Gender**			0.579		0.570
Male	112	65(58.04)		30(26.79)	
Female	38	24(63.16)		12(31.58)	
**Age**			0.541		0.914
≧60	99	57(57.58)		28(28.28)	
<60	51	32(62.75)		14(27.45)	
**Tumor size**			0.567		0.005**
T1-2	119	72(60.50)		27(22.69)	
T3-4	31	17(54.84)		15(48.39)	
**Stage**			0.013*		0.003**
I-II	98	51(52.04)		23(23.47)	
III-IV	52	38(73.08)		19(36.54)	
**Differentiation**			0.085		0.012*
Well	34	15(44.12)		9(26.47)	
Moderately	89	54(60.67)		28(31.46)	
Poorly	27	20(74.07)		15(55.56)	
**LN metastasis**			0.039*		0.000**
Negative Positive	96	51(53.13)		15(15.63)	
	54	38(70.37)		27(50.00)	

*P < 0.05, **P < 0.01.

**Figure 3:**
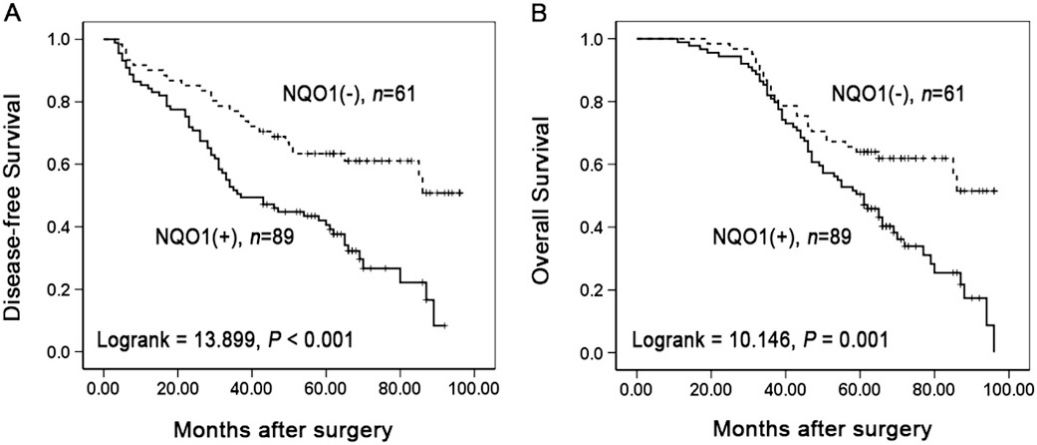
**Kaplan-Meier analysis of DFS and OS rates in 150 NSCLC patients in relation to NQO1 protein expression.** Patients of NSCLC with NQO1 positive expression had lower DFS (**A**, *P* < 0.001) and OS (**B**, *P* < 0.001) rates than those with NQO1 negative expression (+, positive expression; −, negative expression).

To further substantiate the importance of NQO1 expression in NSCLC progression, we analyzed the relationships between NQO1 positive expression rate and DFS and OS in 150 lung cancer cases using the Kaplan-Meier method, and found that patients with NQO1 positive expression had lower DFS (Log-rank = 13.899, *P* < 0.001) and OS (Log-rank = 10.146, *P* = 0.001) rates than those with NQO1 negative expression (Figure 3A & B).

**Figure 4:**
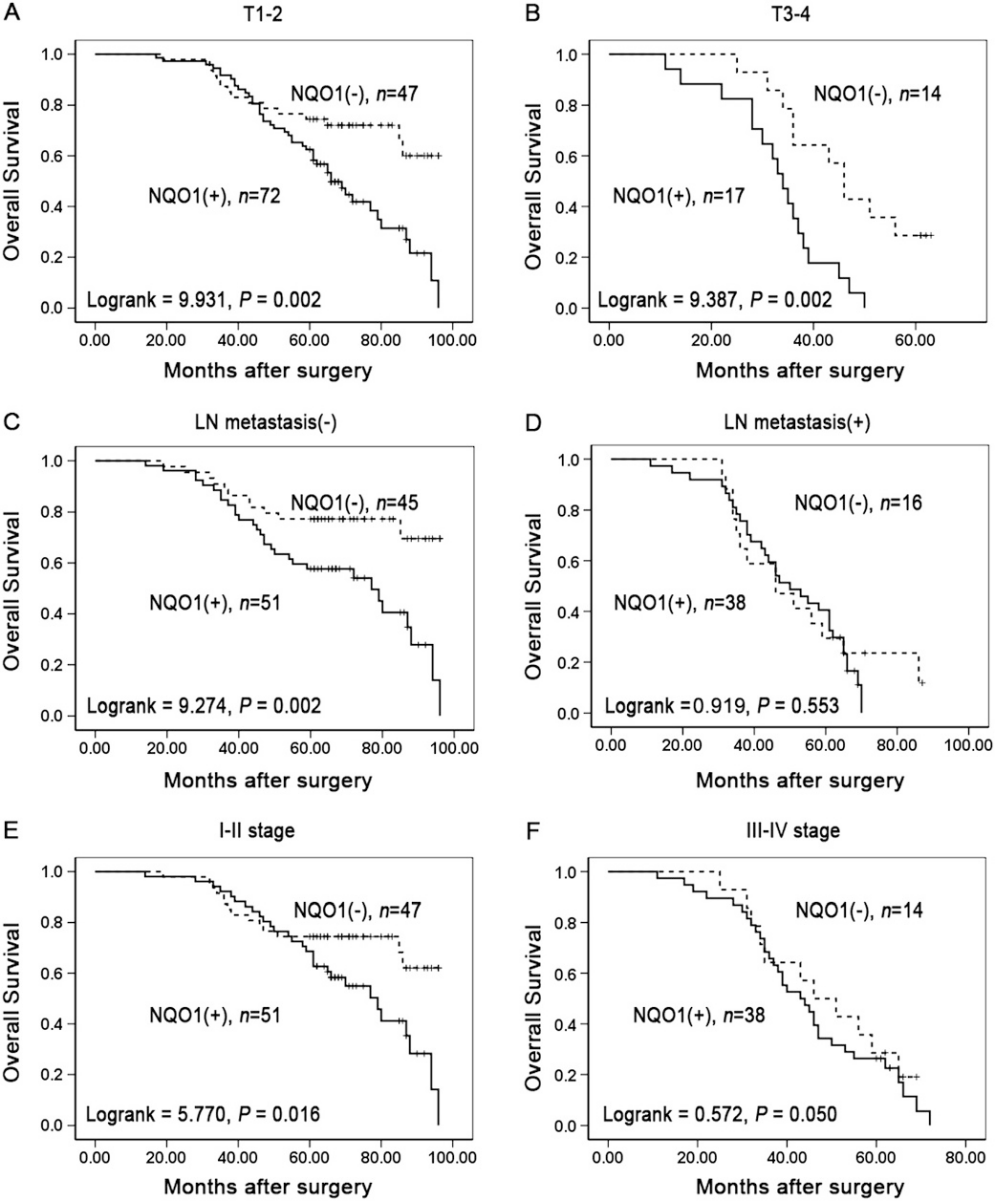
**Kaplan-Meier analysis of OS rates in patients with or without NQO1 expressed NSCLC in prognostic factors.** OS was assessed in NSCLC patients with T1-2 (**A**, *P* = 0.002), T3-4 (**B**, *P* = 0.002), LN metastasis (−) (**C**, *P* = 0.002), LN metastasis (+) (**D**, *P* = 0.553), I-II stage (**E**, *P* = 0.016), and III-IV stage (**F**, *P* = 0.050) concomitant with either positive- or negative-expression of NQO1.

Similarly, we also analyzed the association between the NQO1 expression and tumor size, lymph node metastasis, and clinical stages of NSCLC. The patients with NQO1 positive expression had lower OS rates compared with those with NQO1 negative expression in the groups of T1-2 (Log-rank = 9.931, *P* = 0.002), T3-4 (Log-rank = 9.387, *P* = 0.002) (Figure 4A & B), without LN metastasis (Log-rank = 9.274, *P* = 0.002) and stage I-II of NSCLC (Log-rank = 5.770, *P* = 0.016) (Figure 4C & E), however, in the groups of patients with LN metastasis or III-IV stages, OS rate was not correlated with NQO1 expression status (Log-rank = 0.919, *P* = 0.553 and Log-rank = 0.572, *P* = 0.050, respectively) (Figure 4D & F).

### NQO1 expression is an independent prognostic biomarkerin NSCLC by Cox proportional hazardsregression model

Univariate analysis demonstrated that the NSCLC patients with NQO1 positive expression had significant lower OS rate (HR: 1.442, 95% CI: 1.036-2.007, *P* = 0.030) than those with NQO1 negative expression. Additionally, age (HR: 1.498, 95% CI: 1.062-2.113, *P* = 0.021), tumor size (HR: 5.566, 95% CI: 3.499-8.857, *P* = 0.000), differentiation (HR: 1.426, 95% CI: 1.101-1.847, *P* = 0.007), lymph node metastasis (HR: 2.300, 95% CI: 1.607-3.292, *P* = 0.000)and clinical stage (HR: 3.720, 95% CI: 2.526-5.477, *P* = 0.000) were all significantly associated with OS rates of NSCLC patients. Then, multivariate analysis was performed using the Cox proportional hazards model for all of the significant variables, which were examined in the univariate survival analysis. We found that NQO1 expression emerged as a significant independent prognostic factor for OS rates in patients with NSCLCs (HR: 1.514, 95% CI: 1.066-2.151, *P* = 0.020) along with tumor size (HR: 5.545, 95% CI: 3.283-9.366, P = 0.000), differentiation (HR: 1.369, 95% CI: 1.055-1.775, *P* = 0.018), lymph node metastasis (HR: 1.962, 95% CI: 1.334-2.884, *P* = 0.001) and clinical stage (HR: 2.192, 95% CI: 1.403-3.425, *P* = 0.001) (Table 3).

**Table 3:** **Univariateand multivariate analysis of clinicopathological factors for the overall survival rate of 150 patients with NSCLC**

Characteristics	Univariate analysis HR (95% CI)	*P*value	Multivariate analysis HR (95% CI)	*P*value
**Gender**	1.047(0.724-1.513)	0.809	1.023(0.700-1.496)	0.907
**Age**	1.498(1.062-2.113)	0.021*	1.050(0.728-1.516)	0.793
**Tumor size**	5.566(3.499-8.857)	0.000**	5.545(3.283-9.366)	0.000**
**Differentiation**	1.426(1.101-1.847)	0.007**	1.369(1.055-1.775)	0.018*
**LN metastasis**	2.300(1.607-3.292)	0.000**	1.962(1.334-2.884)	0.001**
**Stage**	3.720(2.526-5.477)	0.000**	2.192(1.403-3.425)	0.001**
**NQO1**	1.442(1.036-2.007)	0.030*	1.514(1.066-2.151)	0.020*

LN: lymph node; HR: hazard ratio; CI: confidence interval.

*P <0.05, **P <0.01.

## Discussion

NQO1, known as NAD(P)H: quinone oxidoreductase-1, was first identified by Ernster and Navazio in 1958 [14]. NQO1 is a homodimericflavoprotein and many functions have been proposed, such as xenobiotic detoxification, superoxide scavenging, modulation of p53, maintenance of endogenous antioxidants, and proteasomal degradation [6]. Several studies have indicated that the phase II enzyme NQO1 catalyzes the metabolic detoxification of quinones and protects cells against chemical-induced oxidative stress and cancer [15,16]. Nagata *et al.* [17] and Malik *et al.* [18] reported that the C609T polymorphism in the NQO1 gene affects the translation of the NQO1 protein, and have been reported to be associated with an increased risk of cancers death. Moreover, NQO1 polymorphism that leads to the enzyme inactivity has been found to be a strong prognostic and predictive factor in the poor outcome of breast cancer [19]. NQO1 has also been shown to act as a chaperone, thereby stabilizing various proteins, including the tumor suppressor protein p53 [20] and other short-lived proteins such as ornithine decarboxylase [21]. These studies suggested that NQO1 activities may be essential for cancer progression.

Accumulating studies showed that NQO1 was expressed at relatively high levels in many solid tumors. For example, our previous study [22] demonstrated that NQO1 protein expression was significantly elevated in breast cancer tissues compared with hyperplasia or adjacent non-tumor tissues, indicating that NQO1 up-regulation may occur in the initiation stage of breast cancer progression. Similarly, compared with normal cervical epithelia, the strongly positive rate of NQO1 protein expression was also significantly higher in cervical SCC and intraepithelial neoplasia tissues, indicating that NQO1 expression might be related to tumorigenesis of cervical cancer [23]. Furthermore, we also found that NQO1 protein was frequently high-expressed in gastric adenocarcinoma compared with the gastric dysplasia and adjacent non-tumor tissues, indicating that NQO1 was a significant prognostic or predictive maker of gastric adenocarcinoma [24]. Consistently, Awadallah *et al.* [25] and Lyn-Cook *et al.* [26] reported that NQO1 protein was up-regulated in pancreatic ductal adenocasinoma, and also considered that NQO1 may represent a role of useful biomarker for pancreatic cancer. Malkinson *et al.* [27] found that NQO1 gene was observed to be high-expressed in human lung cancer tissues, and Rosvold *et al.* [28] and Heller *et al.* [29] also indicated that the gene encoding NQO1 is a promising candidate in the pathogenesis of lung cancer. However, to date, the clinicopathological significance of NQO1 protein expression in NSCLC has not been elucidated.

Thus, here we performed IF and IHC staining in 150 NSCLC paired with the adjacent non-tumor tissues and 14 normal lung tissues, and found that NQO1 protein localized in the cytoplasm of A549 lung cancer cells and NSCLC tissues. Both positive and strongly positive rates of NQO1 protein expression were significantly higher than both in adjacent non-tumor and normal lung tissues. These results indicate that NQO1 played an important role in the progression of lung cancer. Mikami K *et al.* [30] reported that the expression and enzyme activity of NQO1 was up-regulated in colon cancer cell lines and colorectal tumors, and moreover significantly higher in tumors with LN metastases than those without metastasis. Here we analyzed the correlation between NQO1 expression and clinicopathological parameters of NSCLC, and the results showed that NQO1 expression and high-expression was all significantly associated with LN metastasis and clinical stage. Moreover, the strongly positive rate of NQO1 protein was higher in NSCLCs with larger tumor size (>5 cm) than in cases with smaller (≤5 cm), and it was also significantly higher in poorly differentiated NSCLC than in moderately and well differentiated NSCLC. These results indicated that NQO1 might be a predictive biomarker for poor prognostic evaluation of NSCLCs, and NQO1 protein maybe participated in the tumorigenesis and malignant progression of NSCLC.

In regard to survival, we previously found that high expression of NQO1 protein was strongly associated with advanced stage, lymph node metastasis, Her2 overexpression and shortened survival of patients with breast cancer [22]. Moreover, Buranrat *et al.* [12] also reported a significant association between high level of NQO1 expression and short overall survival time of cholangiocarcinoma patients, which raised the exciting possibility of using NQO1 as a tumor marker. However, Kim *et al.* [31] reported that there was no correlation between NQO1 and prognosis of small-cell lung cancer. Here we found that NSCLC patients with NQO1 protein positive-expression had a lower DFS and OS rates than those with NQO1 protein negative-expression. Additionally, age, tumor size, differentiation, lymph node metastasis, clinical stage and NQO1 expression were all significantly associated with OS rates of NSCLC patients (*P* < 0.05). Furthermore, multivariate survival analysis demonstrated that NQO1 positive-expression was an independent prognostic factor along with tumor size, differentiation, lymph node metastasis and clinical stage. These findings indicated that NQO1 might be a potentially predictive biomarker of poor prognosis, especially in patients with poor differentiation, lymph node metastasis and clinical stage of NSCLC.

Recently, NQO1 has been used as the target enzyme in tumor cells to exemplify the ‘enzyme directed’ approach to anticancer drug development [32]. Park *et al.* [33] and Kung *et al.* [34] demonstrated that NQO1 bioactivatable drugs (β-Lapachone or deoxynyboquinone [DNQ]) can effectively kill the cancer cells. Huang *et al*. [35] reported that the potency and NQO1-dependent therapeutic window of DNQ and its apparent reduced metabolism by one-electron oxidoreductases make this drug (or derivatives) very promising. Therefore, the further study will be significant to verify if the NQO1 inhibitor could be used for the therapy of patients with NSCLC.

## Conclusions

In conclusion, NQO1 is frequently upregulated in NSCLC, and it may be a useful poor prognostic biomarker and a potential therapeutic target for patients with NSCLC.

## Abbreviations

NQO1: NAD(P)H quinone oxidoreductase 1
NSCLC: Non-small cell lung cancer
SCC: Squamous cell carcinoma
EGFR: Epidermal growth factor receptor
EML4-ALK: Echinoderm microtubule associated protein like4-anaplastic lymphoma kinase
DNQ: Deoxynyboquinone
